# Age-Related Differences in Emotional Responses Among Individuals with Multiple Sclerosis

**DOI:** 10.1093/geroni/igaf122.3211

**Published:** 2025-12-31

**Authors:** Kyungmi Lee, Matthew Plow

**Affiliations:** Case Western Reserve University, Cleveland, Ohio, United States; Case Western Reserve University, Cleveland, Ohio, United States

## Abstract

Socioemotional theories explain that despite physical decline, psychological changes in aging occur due to adaptation. Aging-related psychological changes may impact emotional responses, such as happiness, stress, and fatigue. However, research on how age affects these factors in individuals with multiple sclerosis (MS) is limited. This study examines how aging affects emotional responses (i.e., happiness, stress, and fatigue) by comparing individuals with MS who are under 55 and over 55, focusing on age-related differences in emotional responses over time. Data from 107 MS participants were collected using ecological momentary assessment, with five daily measurements over 10 days, yielding 6,600 observations. A multilevel model assessed both within-person and between-person differences, controlling for gender and race. In the within-person analysis, previous-day happiness, stress, and fatigue significantly predicted current levels (happiness: β = 0.194, p < 0.001; stress: β = 0.247, p < 0.001; fatigue: β = 0.217, p < 0.001). Weekly patterns showed increasing happiness toward the end of the week (β = 0.106, p < 0.01), decreasing stress (β = -0.213, p < 0.01), and mid-week fatigue slightly increased (β = 0.075, p < 0.05). Although between-person differences were not statistically significant, younger adults showed greater emotional fluctuations (happiness: β = 0.220 vs. β = 0.160; stress: β = 0.260 vs. β = 0.230; fatigue: β = 0.230 vs. β = 0.060). Aging in MS may be associated with reduced emotional variability due to adaptive mechanisms in aging. Further research is needed to explore the mechanisms and implications for age-specific MS interventions.

